# A Review of the Clinical Outcomes for Patients Diagnosed with Brainstem Metastasis and Treated with Stereotactic Radiosurgery

**DOI:** 10.1155/2013/652895

**Published:** 2013-04-11

**Authors:** Andrew F. Lamm, Ameer L. Elaimy, Wayne T. Lamoreaux, Alexander R. Mackay, Robert K. Fairbanks, John J. Demakas, Barton S. Cooke, Christopher M. Lee

**Affiliations:** ^1^Gamma Knife of Spokane, 910 W 5th Avenue, Suite 102, Spokane, WA 99204, USA; ^2^Cancer Care Northwest, Deaconess Health and Education Building, 910 W 5th Avenue, Suite 102, Spokane, WA 99204, USA; ^3^MacKay & Meyer MDs, Spokane, WA 99202, USA; ^4^Spokane Brain & Spine, Spokane, WA 99204, USA

## Abstract

Only 3%–5% of all brain metastases are located in the brainstem. We present a comprehensive review of the clinical outcomes from modern studies that treated patients with brainstem metastasis using either a Gamma Knife or a linear accelerator-based stereotactic radiosurgery. The median survival time of patients was compared to better understand what clinical or treatment factors are predictive of improved survival. This information can then be utilized to optimize patient care. The data suggests that higher prescribed marginal dose and the associated greater local control of brainstem lesions are associated with longer patient survival. Further research is necessary to better describe the most effective dose for individual brainstem lesions and to tailor optimum therapy to specific patient subgroups.

## 1. Introduction

 Brain metastases travel via the vascular system to the brain [1], and 40% of patients diagnosed with systemic cancer will develop brain metastasis [2, 3]. Although the occurrence of BSM is relatively low (approximately 3%–5% of all brain metastasis cases), the prognosis for this subset is very poor [4]. Clinical reviews of patients treated for BSM report historical median survival times ranging from 1 to 11 months [5]. Furthermore, the delicate nature and location of the brainstem make it a difficult structure to treat. There is excessive morbidity from conventional neurosurgery and most chemotherapeutic agents are ineffective secondary to the blood-brain barrier. 

Whole-brain radiation (WBRT) therapy and stereotactic radiosurgery (SRS) (by Gamma Knife or linear accelerator [LINAC]) have proved to be effective treatment modalities for BSM [4–10]. In fact, for many patients diagnosed with BSM, radiation is the only viable treatment modality. Both LINAC and Gamma Knife treatments expose cancerous lesions to high energy X-rays in an attempt to cause irreversible damage to the DNA strands of the target cells and eventually cause cell death. 

The purpose of this paper is to review the clinical outcomes and survival statistics of patients diagnosed with brainstem metastasis who have been treated by these targeted radiation techniques.

## 2. Review 

Due to the difficulty and increased risk of treating BSM with conventional neurosurgery, treatment with radiation therapy (especially SRS) has become standard. We review and discuss twelve series published between 1999 and 2011. In each of these studies, patients were treated with SRS using either LINAC- or Gamma-Knife-based systems. 

### 2.1. Survival

As seen from Table 1, patients diagnosed with BSM have median survival times ranging from 4.2 to 12 months. The average median survival time for the data collected in Table 1 was 7.7 months. Fuentes et al. [15] report a median survival of twelve months; the highest median survival of any study to date. Hatiboglu et al. [1] had the lowest reported median survival time at 4.2 months. Some of the variances seen between the median survival statistics of these studies may be due to differences in initial patient Karnofsky performance status (KPS) for each study, frequency of single BSM cases, the number of patients receiving WBRT, intracranial disease burden, and extent of systemic disease, among others. The variability in median survival seen in these studies (see Table 1) may be partially treatment related.

Previous reports of brain metastases, and the subset of those located in the brainstem, find that the absence of active extracranial disease is one of the most important factors influencing median survival [7, 8, 10, 13, 14, 16]. Other factors identified by earlier studies that illustrate a correlation with increased survival include higher KPS scores, lower recursive partitioning analysis (RPA) class, smaller tumor volume, nonmelanoma primary tumor, single metastasis, and higher marginal dose [4, 5, 13, 14]. Furthermore, many studies report that only a small percentage of their patients succumb to progression of intracranial disease after treatment with regimens including SRS [1, 4, 7, 8, 10, 15], supporting the viability of SRS for the treatment of BSM.

### 2.2. KPS and RPA

The Karnofsky performance status (KPS) is a quantified measure of the patients well being and activity, with a score of 100 representing excellent performance status and no signs of disease. A score of zero on the KPS represents patient death. Sneed et al. [17], Hussain et al. [8], Simonova et al. [18], and Lorenzoni et al. [13] assert that KPS scores greater than 70 correlate with increased survival. Yoo et al. [4], Dea et al. [2], and Andrews et al. [19] also report that lower scores on RPA (class I or II) correspond to increased survival. RPA uses a decision tree to classify patients according to different dependent variables and strives to make predictions based upon the classifications. In both cases, better scores on both the KPS and the RPA correlate with longer patient survival.

### 2.3. Tumor Volume, Histology, and Intracranial Disease

It has also been reported that smaller tumor volumes positively correlate with improved survival [5, 14]. Kased et al. [14] claim that tumor volumes less than 1 cm^3^ predict better clinical outcomes. Possible reasons for this include the ability to treat smaller tumors with a greater marginal dose or perhaps that smaller tumors exert a less pressure and distortion on the normal brainstem. Kased et al. [14] also suggest that BSM with a nonmelanoma or nonrenal cell histology predicts longer survival times. These histologies have historically been considered relatively more radioresistant. They tend to require higher doses, which may be more difficult to achieve in the brainstem. Furthermore, Kased et al. [14] state that the absence of other intracranial metastases besides the BSM is significantly associated with longer survival times. Again, further research is necessary to determine the complete explanation for this. One possibility is that the presence of additional intracranial metastasis represents a higher disease burden systematically, which has also been shown to play a significant role in patient survival. Another possibility is that the addition of these intracranial metastases could potentially lower the dose that can be prescribed to each lesion. As the number of intracranial lesions increases, the dosimetric plan becomes more complicated and must account for collisions, normal brain structure safety, and field overlap. From the collected information, a smaller tumor volume, a nonmelanoma or nonrenal cell histology, and an absence of other intracranial metastases correlate with improved survival.

### 2.4. Marginal Dose

Higher marginal doses also show a correlation to improving patient survival time [1, 13, 20]. The group Hatiboglu et al. [1] reported that marginal doses greater than 14 Gy correlated with longer patient survival times. Lorenzoni et al. [13] make the same assertion but report a marginal dose greater than 18 Gy. However, there is some debate regarding optimal dosing schemes as one group, Valery et al. [11], claim that lower marginal doses can achieve similar local control and survival rates as those reported in other clinical studies that used higher dosing schemes. Table 1 illustrates that Valery et al. [11] used a median marginal dose of only 13.4 Gy and achieved a local control rate of 90% and a median survival time of ten months. A more detailed look at Table 1 does not reveal any startling differences between the study of Valery et al. [11] and those of Hatiboglu et al. [1] and Lorenzoni et al. [13]. Additionally, an investigation of the treatment plans and processes does not show any significant differences that may account for this variance. Of course, this is only a crude comparison; however, the general consensus of the medical literature seems to suggest that higher marginal doses are associated with better tumor control and improved patient survival.

### 2.5. Trends Observed in Collected Outcomes

Review of the studies listed in Table 1 reveals intriguing trends. One observation is that there is a general trend between higher marginal prescribed doses and increased median survival times. Although the number of patients in the study cohort who received WBRT differs slightly when compared with dose prescriptions and median survival times, the retrospective nature of the studies and unmatched comparison may contribute additional biases to the trend. Additionally, the variability in tumor volumes of the patients in the studies compared to median survival and prescription does not suggest that the differences seen in the patient survival times are due to differences in patient tumor volumes. With the exception of the group Valery et al. [11], who report longer patient survival with a lower range dose (13.4 Gy), there appear to be a dose response relationship and correlation between longer median survival times and a higher prescription dose. It is imperative that additional research be carried out to more fully understand the relationship between dose and survival.

Another trend seen in Table 1 is the correlation between higher local control and better median survival times. From the information available, local control rates of greater than 90% appear to correspond with increased median survival. The groups Valery et al. [11], Lorenzoni et al. [13], Hussain et al. [8], Fuentes et al. [15], and Huang et al. [7] report local control rates as 90%, 95%, 100%, 92%, and 95%, respectively, and median survival times of 10.0, 11.1, 8.5, 12.0, and 9.0 months, respectively (see Table 1). A report by Vogelbaum et al. [21] evaluated the relationship between local control and prescription dose. They found that at one year local control was 85% for 24 Gy, 49% for 18 Gy, and 45% for 15 Gy. Our review suggests that higher median doses to brainstem tumors are not only tolerable, but also may be preferred for patients who are able to safely receive higher radiation doses (i.e., patients who have not undergone WBRT). It is imperative that additional research be carried out to more fully understand the relationship between dose and survival.

### 2.6. Radiation Limits for the Brainstem

Further research determining the radiation tolerance of the brainstem is needed to better understand the relationship between marginal dose and patient survival. A study by Sharma et al. [22] in 2008 reported that exposing the brainstem to more than 12 Gy at a tumor volume as low as 0.01 cm^3^ could produce new neurological deficits. A review by Mayo et al. [23] in 2010 suggests a similar dose limit of 12.5 Gy to the brainstem. However, this group acknowledges that higher doses (15–20 Gy) have been used on the brainstem with low complication rates and that further research is needed.

 The two previously-mentioned studies suggest low radiation limits for the brainstem. However, none of the studies in this review report a mean marginal dose of less than 13 Gy (see Table 1). Lorenzoni et al. [13], Fuentes et al. [15], and Yen et al. [10] report mean marginal doses of 20 Gy, 19.6 Gy, and 17.6 Gy, respectively. Furthermore, these groups report zero complications and the highest patient median survival times of any study to date (see Table 1). This implies that the brainstem may be more tolerant of higher single fraction doses than previously thought and that there may be a benefit in choosing a higher marginal dose for SRS treatment.

### 2.7. Complications

Unfortunately, WBRT and SRS may include potential side effects such as headache, fatigue, nausea, vomiting, necrosis, neurologic deficits, and edema. The studies incorporated in this paper report a variety of complications due to treatment (see Table 2). Yoo et al. [4] reported one complication, a man who died of hemorrhagic tumor increase as a result of Gamma Knife SRS to a pontine deposit treated at 14.8 Gy. Valery et al. [11] stated that four of their patients had acute headaches after LINAC-based SRS, but were effectively treated with corticosteroids. Hatiboglu et al. [1] report that twelve of their patients developed fifteen complications due to LINAC-based SRS with serious neurological deficits occurring in two patients (hemiparesis in one patient and hemiparesis and cranial nerve deficit with hemorrhage in another). Kelly et al. [12] assert that two patients acquired RTOG grade three toxicities in response to LINAC based SRS, confusion in one and ataxia in the other. Gamma Knife SRS performed by Koyfman et al. [5] yielded no grade three or four toxicities; however, weakness, ataxia, and bleeding were observed in three patients. The study by Kased et al. [14], which used a Gamma Knife to deliver SRS, claimed that four patients developed complications. However, they also found that there was no correlation between prescription dose and complication. Hussain et al. [8] report one complication following SRS with Gamma Knife, in which the patient developed a right-sided hemiparesis that persisted despite treatment with corticosteroids. The review by Shuto et al. [9] stated that two patients treated with Gamma Knife SRS developed complications. Huang et al. [7] reported seven complications after SRS was performed with the Gamma Knife. Four of these seven patients experienced postoperative nausea, vomiting, or dizziness that resolved within hours. The other three patients had seizures. Finally, as mentioned previously, the reports by Lorenzoni et al. [13], Fuentes et al. [15], and Yen et al. [10] did not record any SRS-induced complications. Although radiation-induced complications may occur after treatment with a Gamma Knife or LINAC, the frequency is generally low, and the benefits of the treatment far outweigh the risks for many patients [24]. 

Patients diagnosed with brainstem lesions, even lesions of small volume, generally present with significant neurological defects including acoustic and vision-related sensory problems, motor weakness, and altered mental status [9]. If left untreated patients diagnosed with BSM have a median survival of only one month and a likely reduction in their quality of life [25, 26]. Gamma Knife and LINAC treatment modalities provide a safe and effective way to treat BSM, which in turn may reduce neurological defects and increase patient survival. 

### 2.8. Selecting Patients for Treatment

In selecting patients for WBRT, SRS, or a combination of both, it is important to consider the number of brain metastases, the KPS score, the location of the lesion(s), the age of the patient, the extent of extracranial disease, and the dose that can be safely delivered [7, 10, 13, 27]. Patients who have low number of brain metastases, high KPS scores, lesions in or near delicate regions of the brain (i.e., brainstem, primary motor cortex, etc.), and controlled systemic disease are strong candidates for SRS. Often, patients who have a greater number of brain metastases are candidates for WBRT, sometimes in conjunction with SRS.

## 3. Conclusion

 We present a comprehensive review of the clinical outcomes of modern studies that treated patients with BSM using SRS performed on Gamma Knife and LINAC platforms. Based on the cumulative information compiled from these studies, trends relating to improved patient survival were observed. Higher marginal dose and the resulting improved local control appear to result in improved median survival. There appear to be other factors that influence patient survival as well including the absence of active extracranial disease, higher KPS scores, lower RPA class, smaller tumor volume, nonmelanoma primary tumor, single metastasis, and higher marginal dose. The trends observed in this comprehensive review should be further researched, especially the trend seen between higher marginal dose and median survival time. Based on this review, a relatively safe single fraction marginal dose range for brainstem metastases is 13–21 Gy. Further studies will help clinicians to better understand optimal dosing plans and find the best therapeutic ratio for each individual patient.

## Figures and Tables

**Table 1:** Collected experience of stereotactic radiosurgery for brainstem metastasis.

Study	Year	Method	No. of patients/ No. of lesions	Mean KPS	Median age (yr)	Single BSM*	No. of patients with WBRT	Mean tumor volume (cm^3^)	Mean follow-up (mo)	Median survival (mo)	Mean marginal dose (Gy)	Crude local control (%)
Yoo et al. [4]	2011	GK	32/NR	NR	56$\overset{-}{x}$	6/19%	NR	1.5	12.0	7.7	15.9	87
Valery et al. [11]	2011	LINAC	30/43	80	57$\overset{-}{x}$	20/67%	8/27%	2.8$\tilde{x}$	11.0	10.0	13.4	90
Hatiboglu et al. [1]	2011	LINAC	60/NR	90$\tilde{x}$	61	NR	15/25%	1.0$\tilde{x}$	12.8	4.2	15.0$\tilde{x}$	76
Kelly et al. [12]	2011	LINAC	24/NR	80$\tilde{x}$	57	3/13%	23/96%	0.2$\tilde{x}$	6.6$\tilde{x}$	5.3	13.0	79
Koyfman et al. [5]	2010	GK	43/43	80$\tilde{x}$	59	43/100%	34/79%	0.37$\tilde{x}$	5.3$\tilde{x}$	5.8	15.0$\tilde{x}$	85
Lorenzoni et al. [13]	2009	GK	25/27	79	53	6/24%	17/68%	0.60	5.2	11.1	20.0	95
Kased et al. [14]	2008	GK	42/44	90$\tilde{x}$	55	5/12%	24/57%	0.26$\tilde{x}$	6.9$\tilde{x}$	9.0	16.0$\tilde{x}$	85
Hussain et al. [8]	2007	GK	22/25	92	60	19/86%	3/14%	0.90$\tilde{x}$	8.5$\tilde{x}$	8.5	16.0$\tilde{x}$	100
Fuentes et al. [15]	2006	GK	28/NR	80	58$\overset{-}{x}$	20/71%	6/21%	2.1	11.0$\tilde{x}$	12.0	19.6	92
Yen et al. [10]	2006	GK	53/NR	80	57$\overset{-}{x}$	19/36%	21/40%	2.8	9.8	11.0	17.6	NR
Shuto et al. [9]	2003	GK	25/31	NR	54	8/32%	7/28%	2.1	5.2	4.9	13.0	77
Huang et al. [7]	1999	GK	26/27	80	61	11/42%	24/92%	2.0	9.5$\tilde{x}$	9.0	16.0$\tilde{x}$	95

GK: Gamma Knife, LINAC: linear accelerator, NR: not reported $\overset{-}{x}$ = median, $\tilde{x}$ = mean.

**Table 2:** Radiation-induced complications.

Study	No. of patients with complications	Complication type
Yoo et al. [4]	1	Hemorrhagic tumor increase
Valery et al. [11]	4	Acute headache
Hatiboglu et al. [1]	12	Hemiparesis, hemiparesis with cranial nerve deficit, and hemorrhage
Kelly et al. [12]	2*	Confusion, ataxia
Koyfman et al. [5]	3	Weakness, ataxia, bleeding
Lorenzoni et al. [13]	0	NA
Kased et al. [14]	4	NR
Hussain et al. [8]	1	Right-sided hemiparesis
Fuentes et al. [15]	0	NA
Yen et al. [10]	0	NA
Shuto et al. [9]	2	NR
Huang et al. [7]	7	Nausea, vomiting, dizziness, seizures

*Only reported RTOG Grade III and IV toxicities.

NR indicates not reported.
